# Supplementary material for: A sequential methodology for the rapid identification and characterization of breast cancer-associated functional SNPs
Source: Nat Commun. 2020 Jul 3;11:3340. doi: 10.1038/s41467-020-17159-8 (PMC7334201; doi:10.1038/s41467-020-17159-8)
Supplement: Supplementary file 1 — Supplementary Information [file 41467_2020_17159_MOESM1_ESM.pdf]

Supplementary Information

A sequential methodology for the rapid identification and characterization of breast cancer-associated functional SNPs

Yihan Zhao, Di Wu, Danli Jiang, Xiaoyu Zhang, Ting Wu, Jing Cui, Min Qian, Jean Zhao, Steffi Oesterreich, Wei Sun, Toren Finkel and Gang Li

Supplementary Figure 1 and 2

Supplementary Tables 1-4

Supplementary Figure 1. Dot plot of 3436 SNPs in the function of *Slope* (x axis) versus *P* value (y axis).

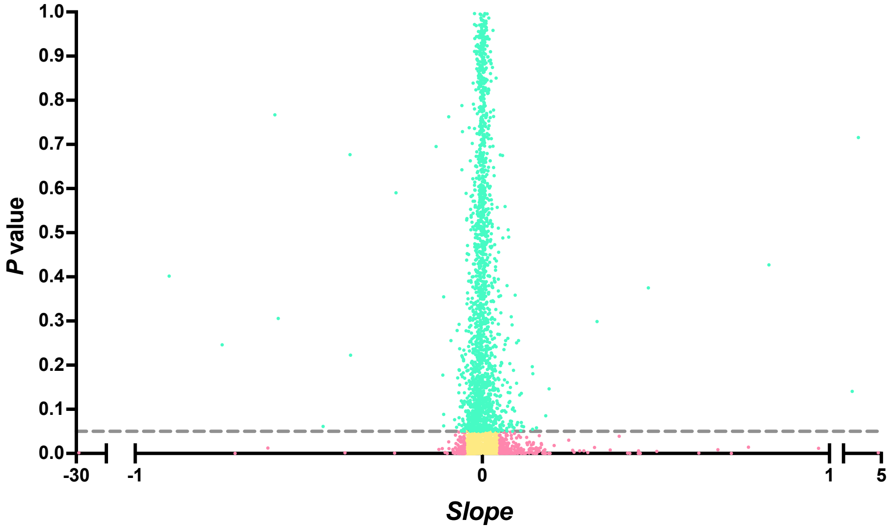

P value for HTP Reel-seq screen was calculated with 5 technical replicates using Student's t-test with 2 tails. Source data are provided as a Source Data file.

Supplementary Figure 2. Regulation of *FGFR2* expression by OCT-1 (A), RUNX2 (B) and FOXA1 (C) via rs2981578.

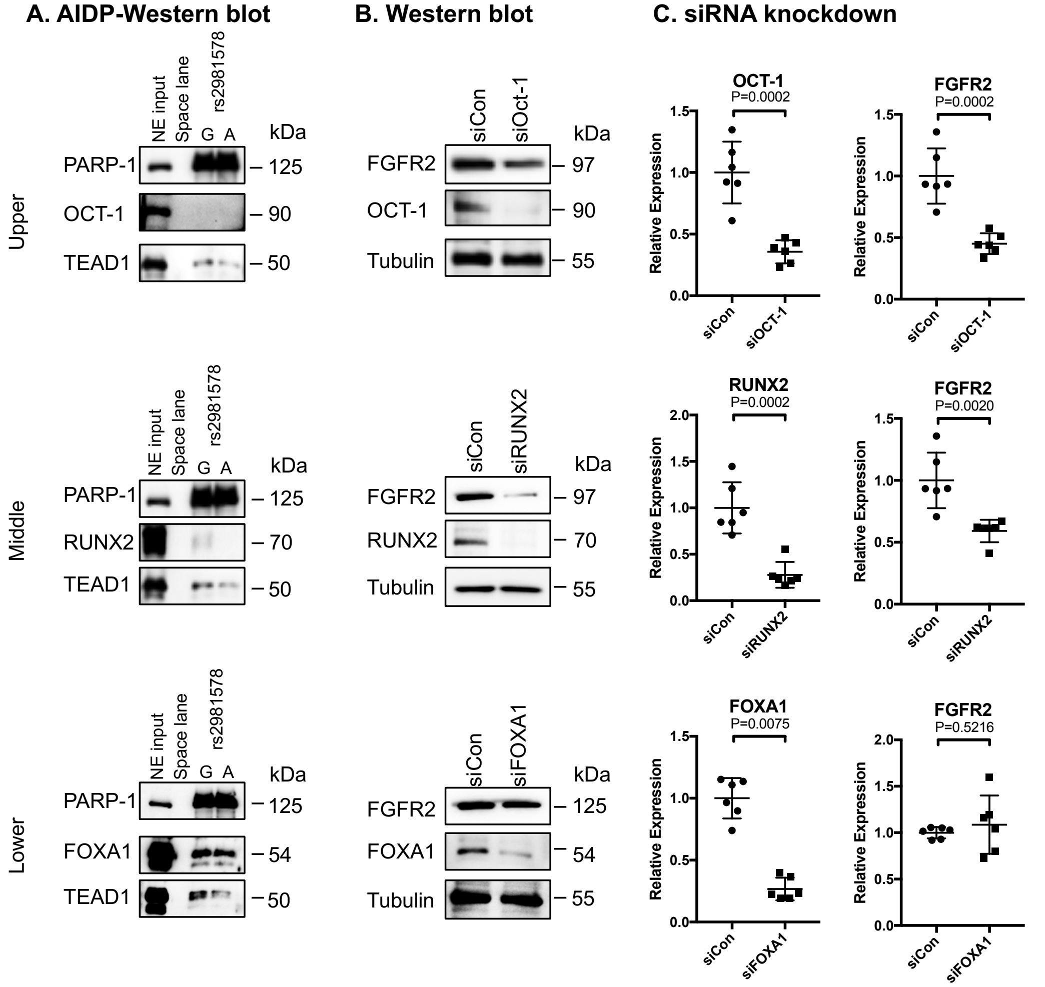

A. AIDP-Western blot showing the no binding of OCT-1 (Upper), allele-imbalanced binding of RUNX2 (Middle), and allele-balanced binding of FOXA1 (Lower) to the two allele of rs2981578 (n=3 biological independent experiments). NE input: nuclear extract control for antibody staining; TEAD1: positive control for allele-imbalanced binding. PARP-1: used for loading control. **B**. Western blot (n=3 biological independendt experiments) and **C**. qPCR (n= 3 biological independendt experiments with each in duplicate) showing the downregulation of FGFR2 expression by OCT-1 (Upper) and RUNX2 (Middle) knockdown and no effect on FGFR2 expression in FOXA1 knockdown cells (Lower). *P* value was calculated using Student's t-test with 2 tails. Error bars in all the plots represent the median with S.E. Source data are provided as a Source Data file.

Supplementary Table 1. Epigenetic and functional annotations of SNPs on the BC-associated *FGFR2* locus.

	SNPs	Promoter histone Marks	Enhancer Histone Marks	DNAse	Proteins bound	Motifs changed	Score	Average
	rs7895676	SKIN	8 tissues			CEBPB,CEBPD,Zbtb3	3	3.6
	rs2981578	5 tissues	13 tissues	11 tissues	FOXA1,HAE2F1	Foxa,Pou2f2,Pou3f2	5	
candidate fSNPs	rs2981584	SKIN	11 tissues	ADRL		4 altered motifs	4	
	rs4752570		13 tissues	5 tissues			2	
	rs1219642	SKIN	BRST, BRN, SKIN			4 altered motifs	4	
	rs2912774	SKIN	7 tissues	LNG		AhR,CTCF	4	3
	rs2912779		13 tissues	7 tissues		AP-4,Pax-6	3	
	rs2981575		7 tissues			Pou5f1	2	
	rs2912780		13 tissues	6 tissues		STAT,VDR	3	
	rs3135718	6 tissues	10 tissues	5 tissues		BRCA1,Hic1	4	
	rs10736303			9 tissues		4 altered motifs	2	
	rs1078806			10 tissues		Hltf,Myc	2	
putative fSNP	rs150005405		6 tissues			17 altered motifs	2	
	rs2162540	SKIN, BRN, GI	7 tissues			T3R	3	
	rs2912778	SKIN, GI	12 tissues	7 tissues		4 altered motifs	4	
	rs2912781		7 tissues			5 altered motifs	2	
	rs2981582	SKIN, BRN, GI	9 tissues	LNG		NF-kappaB,ZEB1	4	
	rs34032268		6 tissues			ZBRK1	2	
	rs35054928	6 tissues	12 tissues	8 tissues	FOXA1,HAE2F1	6 altered motifs	5	
	rs45631539	IPSC	8 tissues			PLAG1,TBX5,Znf143	3	
	rs1219648		7 tissues	IPSC,SKIN,ADRL		Pax-5,THAP1	3	3.2
	rs2936870	BRST, SKIN	7 tissues			13 altered motifs	3	
	rs2860197	SKIN	6 tissues	LNG		FXR,VDR	4	
	rs2420946	SKIN	6 tissues	4 tissues		Zfp161	4	
non-fSNP	rs3135718	6 tissues	10 tissues	5 tissues		BRCA1,Hic1	4	
	rs11200014	SKIN, GI	9 tissues	4 tissues		STAT	4	
	rs2981579		13 tissues	SKIN,PLCNT,SKIN			2	
	rs11599804	4 tissues	13 tissues	4 tissues		AP-2,Ets,RXRA	4	
	rs4752571		8 tissues			4 altered motifs	2	
	rs1219651		BRN			RREB-1	2	

Supplementary Table 2. Primers used in this work

EMSA Primer (5'-3')	
rs7895676-C-F	GCAGTGAGCCCAGATCGCACCACTGCACTCC
rs7895676-C-R	GGAGTGCAGTGGTGCGATCTGGGCTCACTGC
rs7895676-T-F	GCAGTGAGCCCAGATTGCACCACTGCACTCC
rs7895676-T-R	GGAGTGCAGTGGTGCAATCTGGGCTCACTGC
rs2981578-G-F	TCTATGCAAATATGCGGTTTGGAGCAGGGAA
rs2981578-G-R	TTCCCTGCTCCAAACCGCATATTTGCATAGA
rs2981578-A-F	TCTATGCAAATATGCAGTTTGGAGCAGGGAA
rs2981578-A-R	TTCCCTGCTCCAAACTGCATATTTGCATAGA
rs2981584-T-F	TTATGTTTAGTCATCTTGGCTTTGTGCAATA
rs2981584-T-R	TATTGCACAAAGCCAAGATGACTAAACATAA
rs2981584-G-F	TTATGTTTAGTCATCGTGGCTTTGTGCAATA
rs2981584-G-R	TATTGCACAAAGCCACGATGACTAAACATAA
rs1219642-A-F	CCAGGCTGGAGTACAATGGTGCAATTGTACC
rs1219642-A-R	GGTACAATTGCACCATTGTACTCCAGCCTGG
rs1219642-G-F	CCAGGCTGGAGTACAGTGGTGCAATTGTACC
rs1219642-G-R	GGTACAATTGCACCACTGTACTCCAGCCTGG
rs4752570-T-F	CTTCCTGCTGGTGCGTACATGTGCGTATGTG
rs4752570-T-R	CACATACGCACATGTACGCACCAGCAGGAAG
rs4752570-A-F	CTTCCTGCTGGTGCGAACATGTGCGTATGTG
rs4752570-A-R	CACATACGCACATGTTCGCACCAGCAGGAAG
rs4752570-C-F	CTTCCTGCTGGTGCGCACATGTGCGTATGTG
rs4752570-C-R	CACATACGCACATGTGCGCACCAGCAGGAAG
rs16886034-T-F	TAACTAAGCATCTCATAAAAAAGTGCAAAAT
rs16886034-T-R	ATTTTGCACTTTTTTATGAGATGCTTAGTTA
rs16886034-C-F	TAACTAAGCATCTCACAAAAAAGTGCAAAAT
rs16886034-C-R	ATTTTGCACTTTTTTGTGAGATGCTTAGTTA
rs60054381-T-F	AGTGGCTCATGACAGTAATCCCGACACTTTG
rs60054381-T-R	CAAAGTGTCGGGATTACTGTCATGAGCCACT
rs60054381-G-F	AGTGGCTCATGACAGGAATCCCGACACTTTG
rs60054381-G-R	CAAAGTGTCGGGATTCCTGTCATGAGCCACT
rs60054381-A-F	AGTGGCTCATGACAGAAATCCCGACACTTTG
rs60054381-A-R	CAAAGTGTCGGGATTTCTGTCATGAGCCACT
rs74762363-T-F	CAGTTTCAACCACTGTACAAACAGTCAAAAA
rs74762363-T-R	TTTTTGACTGTTTGTACAGTGGTTGAAACTG
rs74762363-A-F	CAGTTTCAACCACTGAACAAACAGTCAAAAA
rs74762363-A-R	TTTTTGACTGTTTGTTCAGTGGTTGAAACTG
rs77371588-T-F	TTTGCTGGGCAGTGATCCCGTCTTCCAGTGG
rs77371588-T-R	CCACTGGAAGACGGGATCACTGCCCAGCAAA
rs77371588-G-F	TTTGCTGGGCAGTGAGCCCGTCTTCCAGTGG
rs77371588-G-R	CCACTGGAAGACGGGCTCACTGCCCAGCAAA
rs111968853-C-F	CCTGACCTTGTGATCCGCCTGCCTCAGCCTC
rs111968853-C-R	GAGGCTGAGGCAGGCGGATCACAAGGTCAGG
rs111968853-T-F	CCTGACCTTGTGATCTGCCTGCCTCAGCCTC
rs111968853-T-R	GAGGCTGAGGCAGGCAGATCACAAGGTCAGG
rs79321361-G-F	GCGCCACGATGCCCAGCTAATTTTGTTTTGT
rs79321361-G-R	ACAAAACAAAATTAGCTGGGCATCGTGGCGC
rs79321361-A-F	GCGCCACGATGCCCAACTAATTTTGTTTTGT
rs79321361-A-R	ACAAAACAAAATTAGTTGGGCATCGTGGCGC
rs8101691-C-F	CTGGCCCAGCACTTCCGGTGGGGTGGCCTTG
rs8101691-C-R	CAAGGCCACCCCACCGGAAGTGCTGGGCCAG
rs8101691-T-F	CTGGCCCAGCACTTCTGGTGGGGTGGCCTTG
rs8101691-T-R	CAAGGCCACCCCACCAGAAGTGCTGGGCCAG

Continue table 2 on next page

Q-PCR Primer (5'-3')	
Q-TEAD1-F	AAGCTCAAACACTTACCAGAGA
Q-TEAD1-R	GGCCATGCAGAGTAGAGTTT
Q-TEAD3-F	GAACAGCGTGCTGGAGAA
Q-TEAD3-R	GTTGGAGACTTCGAAGACAAA
Q-TFAM-F	GTGAGTGACAGTACCCATTATTTC
Q-TFAM-R	ATGAAGAGAAGGAGGGAGAATG
Q-NFIB-F	GCTGTGTCTTATCCAATCCCG
Q-NFIB-R	TGCCTTTGAACAGGATGACCA
Q-PARP2-F	GGCACAAATCAAGGCAGGTTA
Q-PARP2-R	AAGTCATGCGGAATCCTGGTG
Q-FGFR2-F	ACTTGGATCGAATTCTCACTCTC
Q-FGFR2-R	GTGTCAGGGTAACTAGGTGAATAC
Q-GAPDH-F	CGACCACTTTGTCAAGCTCA
Q-GAPDH-R	AGGGGTCTACATGGCAACTG
Q-FOXA1-F	GCAATACTCGCCTTACGGCT
Q-FOXA1-R	TACACACCTTGGTAGTACGCC
Q-RUNX2-F	TCAACGATCTGAGATTTGTGGG
Q-RUNX2-R	GGGGAGGATTTGTGAAGACGG
Q-POU2F1-F	ATGAACAATCCGTCAGAAACCAG
Q-POU2F1-R	GATGGAGATGTCCAAGGAAAGC

Allele-imbalanced DNA Pull Down Western Blot Primer (5'-3') (Reserve same as EMSA)	
Bio-rs7895676-C	/5Biosg/GCAGTGAGCCCAGATCGCACCACTGCACTCC
Bio-rs7895676-T	/5Biosg/GCAGTGAGCCCAGATTGCACCACTGCACTCC
Bio-rs2981578-G	/5Biosg/TCTATGCAAATATGCGGTTTGGAGCAGGGAA
Bio-rs2981578-A	/5Biosg/TCTATGCAAATATGCAGTTTGGAGCAGGGAA
Bio-rs2981584-T	/5Biosg/TTATGTTTAGTCATCTTGGCTTTGTGCAATA
Bio-rs2981584-G	/5Biosg/TTATGTTTAGTCATCGTGGCTTTGTGCAATA

SDCP-MS Primer (5'-3')	
SDCP-rs7895676-C-I	/5Biosg/GTCTGTGTTCCGTTGTCCGTGCTGAATGGATCCGGATCCGCAGTGAGCCCAGATCGCACCACTGCACTCCGAATTCGAATTCGCAC
SDCP-rs7895676-C-I	GTGCGAATTCGAATTCGGAGTGCAGTGGTGCGATCTGGGCTCACTGCGGATCCGGATCCATT
SDCP-rs7895676-T-I	/5Biosg/GTCTGTGTTCCGTTGTCCGTGCTGAATGGATCCGGATCCGCAGTGAGCCCAGATTGCACCACTGCACTCCGAATTCGAATTCGCAC
SDCP-rs7895676-T-I	GTGCGAATTCGAATTCGGAGTGCAGTGGTGCAATCTGGGCTCACTGCGGATCCGGATCCATT
SDCP-rs2981578-G-I	/5Biosg/GTCTGTGTTCCGTTGTCCGTGCTGAATGGATCCGGATCCTCTATGCAAATATGCGGTTTGGAGCAGGGAAGAATTCGAATTCGCAC
SDCP-rs2981578-G-I	GTGCGAATTCGAATTCTTCCCTGCTCCAAACCGCATATTTGCATAGAGGATCCGGATCCATT
SDCP-rs2981578-A-I	/5Biosg/GTCTGTGTTCCGTTGTCCGTGCTGAATGGATCCGGATCCTCTATGCAAATATGCAGTTTGGAGCAGGGAAGAATTCGAATTCGCAC
SDCP-rs2981578-A-I	GTGCGAATTCGAATTCTTCCCTGCTCCAAACTGCATATTTGCATAGAGGATCCGGATCCATT
SDCP-rs2981584-T-I	/5Biosg/GTCTGTGTTCCGTTGTCCGTGCTGAATGGATCCGGATCCTTATGTTTAGTCATCTTGGCTTTGTGCAATAGAATTCGAATTCGCAC
SDCP-rs2981584-T-I	GTGCGAATTCGAATTCTATTGCACAAAGCCAAGATGACTAAACATAAGGATCCGGATCCATT
SDCP-rs2981584-G-I	/5Biosg/GTCTGTGTTCCGTTGTCCGTGCTGAATGGATCCGGATCCTTATGTTTAGTCATCGTGGCTTTGTGCAATAGAATTCGAATTCGCAC
SDCP-rs2981584-G-I	GTGCGAATTCGAATTCTATTGCACAAAGCCACGATGACTAAACATAAGGATCCGGATCCATT

Supplementary Table 3. Antibodies, siRNAs and shRNAs used in this work

Antibody	Manufacturer	Cat#	Dilution	Source
TEAD1	ABCLONAL	A6768	1：1000	Rabbit
TEAD3	ABCLONAL	A7454	1：1000	Rabbit
TFAM	ABCLONAL	A13552	1：1000	Rabbit
NFIB	ABCLONAL	A15294	1：1000	Rabbit
PARP2	ABCLONAL	A16475	1：1000	Rabbit
FGFR2	LifeSpan Biosciences	LS-C172060	1：1000	Rabbit
Ku86(C-20)	Santa Cruz	sc-1484	1：500	Goat
PARP-1(H-250)	Santa Cruz	sc-7150	1：500	Rabbit
α-Tublin	Sigma	T6074	1：1000	Mouse
m-IgGκ BP-HRP	Santa Cruz	SC-516102	1：5000	Mouse
mouse anti-rabbit IgG-HRP	Santa Cruz	SC-2357	1：5000	Mouse
donkey anti-goat IgG-HRP	Santa Cruz	SC-2020	1：5000	Donkey
FOXA1	ABCLONAL	A15278	1：1000	Rabbit
RUNX2	ABCLONAL	A2851	1：1000	Rabbit
POU2F1	ABCLONAL	A1682	1：1000	Rabbit
**shRNA lentivirus**	**Accession number**	**TRC number**	**Target Region**	**Manufatory**
TEAD1	NM_021961	TRCN0000297070	3UTR	sigma
TEAD3	NM_003214	TRCN0000015948	3UTR	sigma
TFAM	NM_003201	TRCN0000016093	3UTR	sigma
NFIB	NM_005596	TRCN0000274124	3UTR	sigma
PAPR2	NM_005484	TRCN0000235599	CDS	sigma
**siRNA**	**Cat**	**Manufatory**		
HUMAN PARP2 siRNA	J-010127-05-0002	Horizondiscovery		
HUMAN TEAD1 siRNA	J-012603-07-0002	Horizondiscovery		
HUMAN TEAD3 siRNA	J-012604-05-0002	Horizondiscovery		
HUMAN TFAM siRNA	J-019734-05-0002	Horizondiscovery		
HUMAN TFAM siRNA	J-019734-05-0002	Horizondiscovery		
HUMAN NFIB siRNA	J-008456-05-0002	Horizondiscovery		
HUMAN FOXA1 siRNA	J-010319-05-0002	Horizondiscovery		
HUMAN RUNX2 siRNA	J-012665-07-0002	Horizondiscovery		
HUMAN POU2F1 siRNA	J-019689-06-0002	Horizondiscovery		

Supplementary Table 4. Primer used in this work for luciferase reporter assay

Luciferase report assay primer (5'-3')	
Luci-rs1219642-A-F	CCCAGGCTGGAGTACAATGGTGCAATTGTACCC
Luci-rs1219642-A-R	TCGAGGGTACAATTGCACCATTGTACTCCAGCCTGGGAGCT
Luci-rs1219642-G-F	CCCAGGCTGGAGTACAGTGGTGCAATTGTACCC
Luci-rs1219642-G-R	TCGAGGGTACAATTGCACCACTGTACTCCAGCCTGGGAGCT
Luci-rs4752570-T-F	CCTTCCTGCTGGTGCGTACATGTGCGTATGTGC
Luci-rs4752570-T-R	TCGAGCACATACGCACATGTACGCACCAGCAGGAAGGAGCT
Luci-rs4752570-A-F	CCTTCCTGCTGGTGCGAACATGTGCGTATGTGC
Luci-rs4752570-A-R	TCGAGCACATACGCACATGTTCGCACCAGCAGGAAGGAGCT
Luci-rs4752570-C-F	CCTTCCTGCTGGTGCGCACATGTGCGTATGTGC
Luci-rs4752570-C-R	TCGAGCACATACGCACATGTGCGCACCAGCAGGAAGGAGCT
Luci-rs16886034-T-F	CTAACTAAGCATCTCATAAAAAAGTGCAAAATC
Luci-rs16886034-T-R	TCGAGATTTTGCACTTTTTTATGAGATGCTTAGTTAGAGCT
Luci-rs16886034-C-F	CTAACTAAGCATCTCACAAAAAAGTGCAAAATC
Luci-rs16886034-C-R	TCGAGATTTTGCACTTTTTTGTGAGATGCTTAGTTAGAGCT
Luci-rs60054381-T-F	CAGTGGCTCATGACAGTAATCCCGACACTTTGC
Luci-rs60054381-T-R	TCGAGCAAAGTGTCGGGATTACTGTCATGAGCCACTGAGCT
Luci-rs60054381-G-F	CAGTGGCTCATGACAGGAATCCCGACACTTTGC
Luci-rs60054381-G-R	TCGAGCAAAGTGTCGGGATTCCTGTCATGAGCCACTGAGCT
Luci-rs60054381-A-F	CAGTGGCTCATGACAGAAATCCCGACACTTTGC
Luci-rs60054381-A-R	TCGAGCAAAGTGTCGGGATTTCTGTCATGAGCCACTGAGCT
Luci-rs74762363-T-F	CCAGTTTCAACCACTGTACAAACAGTCAAAAAC
Luci-rs74762363-T-R	TCGAGTTTTTGACTGTTTGTACAGTGGTTGAAACTGGAGCT
Luci-rs74762363-A-F	CCAGTTTCAACCACTGAACAAACAGTCAAAAAC
Luci-rs74762363-A-R	TCGAGTTTTTGACTGTTTGTTCAGTGGTTGAAACTGGAGCT
Luci-rs77371588-T-F	CTTTGCTGGGCAGTGATCCCGTCTTCCAGTGGC
Luci-rs77371588-T-R	TCGAGCCACTGGAAGACGGGATCACTGCCCAGCAAAGAGCT
Luci-rs77371588-G-F	CTTTGCTGGGCAGTGAGCCCGTCTTCCAGTGGC
Luci-rs77371588-G-R	TCGAGCCACTGGAAGACGGGCTCACTGCCCAGCAAAGAGCT
Luci-rs111968853-C-F	CCCTGACCTTGTGATCCGCCTGCCTCAGCCTCC
Luci-rs111968853-C-R	TCGAGGAGGCTGAGGCAGGCGGATCACAAGGTCAGGGAGCT
Luci-rs111968853-T-F	CCCTGACCTTGTGATCTGCCTGCCTCAGCCTCC
Luci-rs111968853-T-R	TCGAGGAGGCTGAGGCAGGCAGATCACAAGGTCAGGGAGCT
Luci-rs79321361-G-F	CGCGCCACGATGCCCAGCTAATTTTGTTTTGTC
Luci-rs79321361-G-R	TCGAGACAAAACAAAATTAGCTGGGCATCGTGGCGCGAGCT
Luci-rs79321361-A-F	CGCGCCACGATGCCCAACTAATTTTGTTTTGTC
Luci-rs79321361-A-R	TCGAGACAAAACAAAATTAGTTGGGCATCGTGGCGCGAGCT
Luci-rs8101691-C-F	CCTGGCCCAGCACTTCCGGTGGGGTGGCCTTGC
Luci-rs8101691-C-R	TCGAGCAAGGCCACCCCACCGGAAGTGCTGGGCCAGGAGCT
Luci-rs8101691-T-F	CCTGGCCCAGCACTTCTGGTGGGGTGGCCTTGC
Luci-rs8101691-T-R	TCGAGCAAGGCCACCCCACCAGAAGTGCTGGGCCAGGAGCT
Luci-rs7895676-C-F	CGCAGTGAGCCCAGATCGCACCACTGCACTCCC
Luci-rs7895676-C-R	TCGAGGGAGTGCAGTGGTGCGATCTGGGCTCACTGCGAGCT
Luci-rs7895676-T-F	CGCAGTGAGCCCAGATTGCACCACTGCACTCCC
Luci-rs7895676-T-R	TCGAGGGAGTGCAGTGGTGCAATCTGGGCTCACTGCGAGCT
Luci-rs2981578-G-F	CTCTATGCAAATATGCGGTTTGGAGCAGGGAAC
Luci-rs2981578-G-R	TCGAGTTCCCTGCTCCAAACCGCATATTTGCATAGAGAGCT
Luci-rs2981578-A-F	CTCTATGCAAATATGCAGTTTGGAGCAGGGAAC
Luci-rs2981578-A-R	TCGAGTTCCCTGCTCCAAACTGCATATTTGCATAGAGAGCT
Luci-rs2981584-T-F	CTTATGTTTAGTCATCTTGGCTTTGTGCAATAC
Luci-rs2981584-T-R	TCGAGTATTGCACAAAGCCAAGATGACTAAACATAAGAGCT
Luci-rs2981584-G-F	CTTATGTTTAGTCATCGTGGCTTTGTGCAATAC
Luci-rs2981584-G-R	TCGAGTATTGCACAAAGCCACGATGACTAAACATAAGAGCT
