# Supplementary figures and images for: A sequential methodology for the rapid identification and characterization of breast cancer-associated functional SNPs
Source: Nat Commun. 2020 Jul 3;11:3340. doi: 10.1038/s41467-020-17159-8 (PMC7334201; doi:10.1038/s41467-020-17159-8)

rs7895676 rs2981578

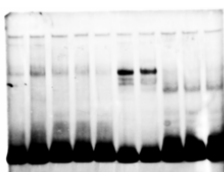

rs2981584

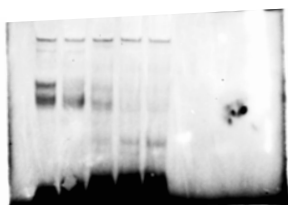

rs4752570

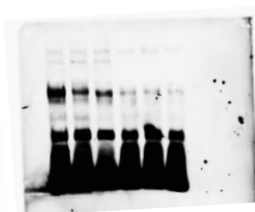

rs1219642

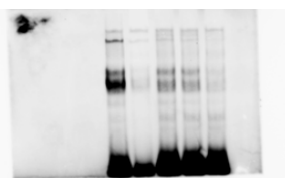

rs16886034

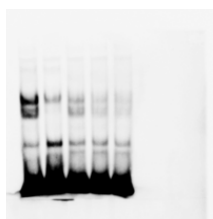

rs60054381

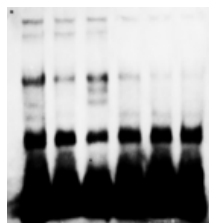

rs74762363

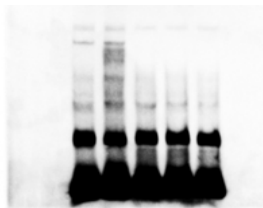

rs77371588

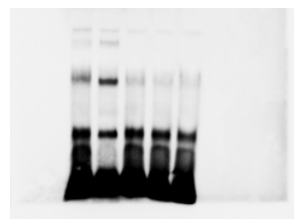

rs79321361

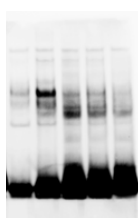

rs8101691

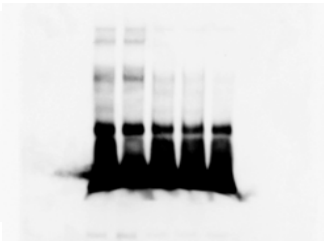

rs111968853

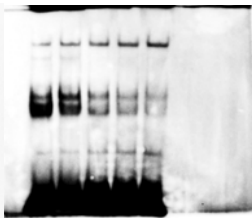

rs2912774

rs2912779

rs2981575

rs2912780

rs3135718

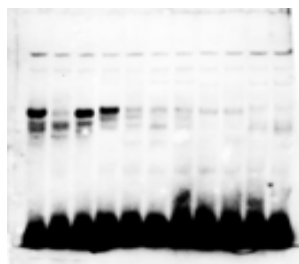

rs10736303

rs1078806

rs150005405

rs2162540

rs2912778

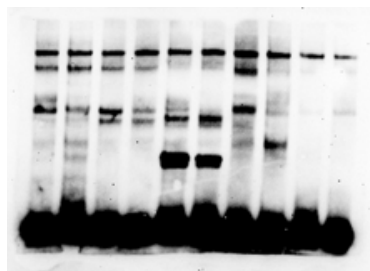

rs2912781

rs2981582

rs34032268

rs35054928

rs45631539

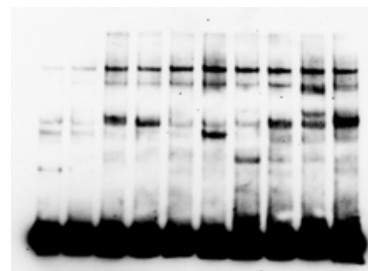

rs1219651

rs2420946

rs2860197

rs2936870

rs4752571

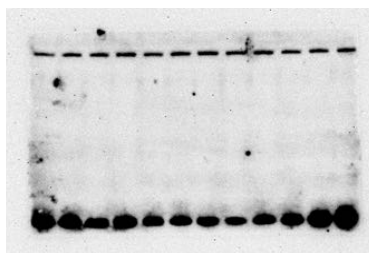

rs11598804

rs11200014

rs2981579

rs1219648

rs3750817

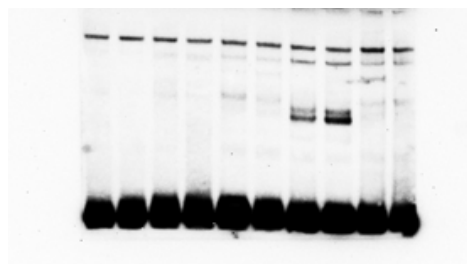

Supplement: Supplementary file 4 — Source Data [file 41467_2020_17159_MOESM4_ESM.zip › Data for Figure 2-3/data-for-Figure-3.pdf]
